# Mining miRNAs' Expressions in Glioma Based on GEO Database and Their Effects on Biological Functions

**DOI:** 10.1155/2020/5637864

**Published:** 2020-10-10

**Authors:** Ke Li, Qi Zhang, Duan Niu, Hailong Xing

**Affiliations:** ^1^Department of Neurosurgery, Binzhou Medical University Hospital, Binzhou 256603, China; ^2^Department of Pediatrics, Binchengqu Shili Hospital, Binzhou 256600, China

## Abstract

**Purpose:**

To mine miR expression in glioma based on the Gene Expression Omnibus (GEO) database and to explore its effects on biological functions.

**Methods:**

Differentially expressed miRs in glioma-related chips were found out based on the GEO database. Fifty patients with glioma treated in our hospital from February 2012 to July 2013 (observation group, OG) and a further 50 healthy people undergoing physical examinations (control group, CG) were enrolled. miR-873-5p expression in serum and in U87, T98G, U251, LN-229, and HEK-293T cells was tested by qRT-PCR. T98G and U251 cells were transfected with miR-873-5p-mimics and miR-NC sequences. The expression in the two cells was also tested by qRT-PCR. The proliferation, invasion, and apoptosis of the transfected cells were, respectively, tested by MTT assay, Transwell, and flow cytometry. The patients were followed up for 5 years to observe their survival.

**Results:**

miR-873-5p expression in OG was remarkably higher than that in CG (*p* < 0.001). miR-873-5p was closely correlated with the tumor diameter, lymph node metastasis, and TNM staging of the patients (*p* < 0.05). According to the plotted receiver operating characteristic (ROC) curves, the areas under the curves (AUCs) of miR-873-5p for diagnosing the disease, tumor diameter, lymph node metastasis, and TNM staging were 0.842, 0.706, 0.865, and 0.793, respectively. The 5-year and recurrence-free survival rates in the low expression group were lower than those in the high expression group. According to multivariate Cox regression analysis, tumor diameter, lymph node metastasis, and miR-873-5p were independent prognostic factors for the disease. After transfection, compared with those in the miR-NC group, T98G and U251 cells in the miR-873-5p-mimic group had remarkably higher miR-873-5p expression (*p* < 0.05), remarkably lower proliferation and invasion rates (*p* < 0.05), and a remarkably higher apoptotic rate (*p* < 0.05).

**Conclusions:**

miR-873-5p can inhibit glioma cells to proliferate and invade, and promote their apoptosis, so it is expected to become a potential diagnostic index and therapeutic target for glioma.

## 1. Introduction

As the most common primary malignant brain tumor in adults, glioma accounts for approximately 70% of these tumors in adults [1], with a high recurrence rate, strong invasiveness, and a poor prognosis [2]. The disease is classified into 4 types according to standards from the World Health Organization (WHO). Glioblastoma multiforme (grade IV), accounting for 65% of all gliomas, has the highest malignancy and the poorest survival, with a one-year survival rate less than 50% [3, 4]. Family heredity and ionizing radiation are two causes of glioma [5], which is treated by surgery, radiotherapy, and chemotherapy at present. Most patients receive surgery first, followed by radiotherapy and chemotherapy to prevent metastasis and recurrence. However, despite various and customized therapeutic methods, glioma is rarely cured, and the median survival time of patients with high-grade glioma is less than 3 years [6, 7]. Continuous surgery and biopsy have certain risks to patients with glioma, and there is currently no biomarker to predict the clinical results and prognosis of the disease. Therefore, to find a crucial biomarker is very important [8].

MicroRNAs (miRs), noncoding short-chain RNAs presented in eukaryotes, regulate cell proliferation, invasion, and apoptosis by regulating target genes [9, 10]. Recent studies have shown that many miRs are abnormally expressed in some cancers and involved in their development and progression, so the miRs have better diagnostic and prognostic values in these cancers [11, 12]. With the development of gene chip technologies and high-throughput sequencing, genes related to diseases have been mined and known through gene expression profiles. The Gene Expression Omnibus (GEO) that covers various biological fields is a public functional genomics data repository, also a global public resource gene expression database established by the National Center for Biotechnology Information (NCBI) [13–15].

Therefore, glioma-related miRs were mined based on the GEO database, and their expression and biological significance were explored, so as to provide reference for clinical practice.

## 2. Materials and Methods

### 2.1. Analysis and Screening of GEO Chips

The GEO database on NCBI (https://www.ncbi.nlm.nih.gov/gds) was logged in, and microRNA glioma was input in the search bar for search. The GSE103228 chip was finally selected. After platform and matrix files of the patients were downloaded, the limma package was used to analyze differentially expressed miRs between normal and cancer tissues, with screening criteria being log FoldChange > 1 and *p* < 0.05. One of the differentially expressed miRs was miR-873-5p. There are currently few studies on miR-873-5p in glioma, so it was explored in this study.

### 2.2. Collection of Clinical Data

Fifty patients with glioma treated in our hospital from February 2012 to July 2013 (OG) were enrolled, including 26 males and 24 females, with an average age of 52.7 ± 6.4 years. Based on the WHO pathological grading, there were 10 cases of grade I, 22 of grade II, 13 of grade III, and 5 of grade IV in OG. Further, 50 healthy subjects undergoing physical examinations in our hospital during the same period (CG) were also enrolled, including 28 males and 22 females, whose average age was 53.4 ± 6.1 years. This study was approved by the Medical Ethics Committee of our hospital. Inclusion criteria for the patients were those diagnosed with glioma by imaging and pathology, those with TNM staging criteria referring to the 7th edition of the American Joint Committee on Cancer Tumor-Node-Metastasis Staging System [16], those with complete clinical data, and those who signed the informed consent form. Exclusion criteria were those who had not received radiotherapy and chemotherapy before, those complicated with other tumors, those with expected survival time greater than 3 months, and those who did not cooperate in follow-up. The differences between the two groups were not statistically significant in their clinical data such as gender and age (*p* > 0.05), which indicated comparability.

### 2.3. Sources of Reagents, Instruments, and Cells

The following are the sources of reagents, instruments, and cells: U87, T98G, U251, LN-229, and HEK-293T cells (Bena Culture Collection, China, BNCC337885, BNCC338721, BNCC100123, BNCC341218, and BNCC341976); Lipofectamine™ 2000 (Invitrogen™, USA, 11668019); a MTT assay kit and a dimethyl sulfoxide (DMSO) reagent (Beyotime Biotechnology, Shanghai, China, C0009, ST038); a Transwell kit, RPMI-1640, phosphate buffer solution (PBS), fetal bovine serum (FBS), and a penicillin-streptomycin double antibody (Gibco, USA, A1142802, 61870044, 14190250, 10437028, and 15070063); trypsin (Thermo Scientific™, 90058); TRIzol (Invitrogen, USA, 15596018); TransScript II Green Two-Step qRT-PCR SuperMix (TransGen Biotech, Beijing, China, AQ202-01 and AQ301-01); an Annexin V/PI apoptosis detection kit (Shanghai Yeasen Biotechnology Co., Ltd., 40302ES20); a microplate reader (BioTek, USA, PerkinElmer); a PCR instrument (ABI, USA, 7500); and a flow cytometer (BD, USA, FACSCanto II). Primer sequences were synthesized by Sangon Biotech (Shanghai) Co., Ltd.

### 2.4. Cell Culture and Transfection

The repurchased U87, T98G, U251, LN-229, and HEK-293T cells were transferred into a culture medium (penicillin-streptomycin double antibody, 10% FBS) and then cultured in a constant temperature incubator at 37°C and with 5% CO_2_. After the miR-873-5p-mimic and miR-NC groups were set up, the cells were transfected using the Lipofectamine™ 2000 kit, with the steps strictly carried out based on the kit instruction. All primers were transfected into the cells with the greatest expression difference.

### 2.5. Detection Methods

#### 2.5.1. qRT-PCR

All subjects' fasting venous blood (5 mL each) was collected, placed in coagulation-promoting tubes, and then centrifuged in a centrifuge (3000 × g at 4°C for 10 min) to collect serum. The cells and serum were collected for total RNA extraction using the TRIzol kit, and its purity, concentration, and integrity were detected by an ultraviolet spectrophotometer and agarose gel electrophoresis. Reverse transcription was performed with 5X TransScript® II All-in-One SuperMix for qPCR and gDNA removal kits, which was strictly conducted based on the manufacturer's kits. Subsequently, PCR amplification was performed, whose system was cDNA (1 *μ*L), upstream and downstream primers (0.4 *μ*L each), 2X TransScript® Tip Green qPCR SuperMix (10 *μ*L), passive reference dye (50x) (0.4 *μ*L), and nuclease-free water finally added to make up to 20 *μ*L. Conditions for the reaction were predenaturation (94°C for 30 s), denaturation (94°C for 5 s), and annealing and extension (60°C for 30 s), and cycling for 40 times. There were 3 similar wells for each sample, and 3 repeated experiments were conducted. With U6 as the internal reference, the data were analyzed by 2^-*ΔΔ*ct^. All primers were provided by Sangon Biotech (Shanghai) Co., Ltd. Primer sequences are shown in Table 1.

#### 2.5.2. Detection of Cell Proliferation

The cells transfected for 24 hours were collected. After the density was adjusted to 3∗10^4^ cells/well, they were inoculated in a 96-well plate for incubation at 37°C for 24, 48, 72, and 96 hours. They were added with 20 *μ*L of MTT solution (5 *μ*g/mL) at each time point and continuously cultured at 37°C for 4 hours. Next, each well was added with 150 *μ*L of DMSO. Finally, the microplate reader was used to measure optical density (OD) values at 570 nm in each group.

#### 2.5.3. Detection of Cell Invasion

The cells transfected for 24 hours were collected. After the density was adjusted to 3∗10^4^ cells/well, the cells were inoculated in a 24-well plate, digested with trypsin, and then transferred to the upper chamber. The upper chamber was added with 200 *μ*L of RPMI-1640 culture solution, while the lower chamber was added with 500 mL of RPMI-1640 (containing 10% FBS), both of which were cultured at 37°C for 48 hours. The matrix and cells that did not pass through the membrane surface in the upper chamber were wiped off. After the Transwell was rinsed with PBS for 3 times and the cells were fixed with paraformaldehyde for 10 min, the upper chamber was cleaned with double distilled water for 3 times, and then stained with 0.5% crystal violet after drying. Cell invasion was observed with a microscope.

#### 2.5.4. Detection of Cell Apoptosis

The cells transfected for 24 hours were firstly digested with 0.25% trypsin and rinsed with PBS for twice. Then, they were added with binding buffer (100 *μ*L), prepared into a 1∗10^6^ cells/mL suspension, successively added with the Annexin V-FITC and PI, and then incubated at room temperature for 5 min in the dark. The FC500MCL flow cytometry system was used for the detection, and the experiment was repeatedly carried out for 3 times to obtain the average value.

### 2.6. Follow-Up

The patients were followed up by telephone and outpatient medical records for 5 years, to record their survival, from the date of admission to their death, their loss or the cut-off date of the follow-up (the day 5 years after the admission). The follow-up contents consisted of the patients' survival status, survival dates, and specific causes of death. The follow-up time was March, June, September, and December each year. Patients with recurrence and metastasis were reviewed by imaging in time to confirm the recurrence and metastasis.

### 2.7. Statistical Analysis

In this study, SPSS20.0 was used to statistically analyze the collected data. GraphPad 7 was used to plot the figures. A K-S test was used to analyze the distribution of measurement data. The data conforming to normal distribution were expressed by mean ± standard deviation (mean ± SD), and the comparison between groups was conducted by independent sample *t* test. The comparison between multiple groups was conducted by one-way analysis of variance (ANOVA) and represented by *F*. Post hoc pairwise comparison was conducted by LSD *t* test. The comparison between multiple time points was conducted by repeated measure ANOVA and represented by *F*. Post hoc test was conducted by Bonferroni. Receiver operating characteristic (ROC) curves were plotted to show the diagnostic value of miR-873-5p in glioma. Multivariate Cox regression analysis was performed on independent risk factors affecting the prognosis. The difference was statistically significant when *p* < 0.05.

## 3. Results

### 3.1. miR-873-5p Had a Diagnostic Value for Glioma and Case Characteristics

**Figure 1:**
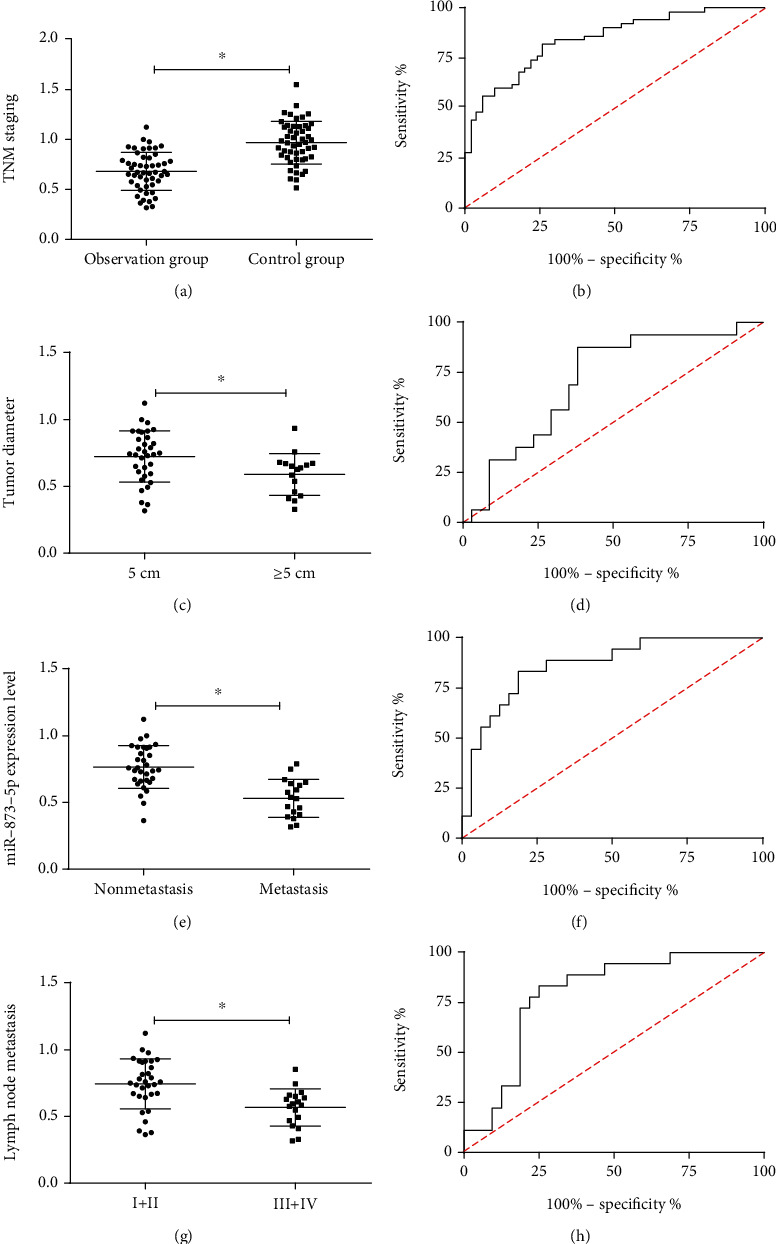
Clinical value of miR-873-5p in glioma. (a) miR-873-5p expression in the patients. (b) The diagnostic value of miR-873-5p for glioma. (c) miR-873-5p expression in the tumor diameter of the patients. (d) The diagnostic value of miR-873-5p for the tumor diameter of the patients. (e) miR-873-5p expression in the lymph node metastasis of the patients. (f) The diagnostic value of *f* miR-873-5p for the lymph node metastasis of the patients. (g) miR-873-5p expression in the TNM staging of the patients. (h) The diagnostic value of miR-873-5p for the TNM staging of the patients. ∗ indicates *p* < 0.05.

According to the detection, miR-873-5p expression in OG was remarkably lower than that in CG (*p* < 0.001). According to the analysis of the relationship between this miR and pathological data, it was closely correlated with the tumor diameter, lymph node metastasis, and TNM staging of the patients (*p* < 0.05). According to the ROC curves, the areas under the curves (AUCs) of miR-873-5p for diagnosing the disease, tumor diameter, lymph node metastasis, and TNM staging were 0.842, 0.706, and 0.865. See Figure 1 and Tables 2 and 3.

### 3.2. Patients with Low miR-873-5p Expression Had Poorer Survival Status

**Figure 2:**
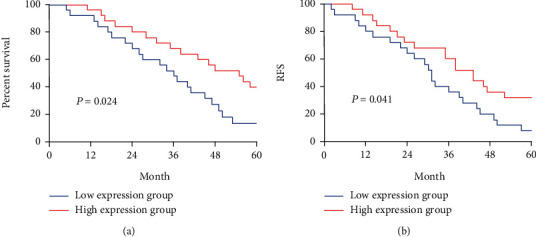
Five-year survival status of patients. (a) The 5-year survival rate in the low expression group was remarkably lower than that in the high expression group (*p* = 0.024). (b) The 5-year RFS in the low expression group was remarkably lower than that in the high expression group (*p* = 0.041).

The 5-year follow-up of the patients was collected, and all patients were followed up with a follow-up rate of 100%. During the 5 years, 36 cases died from the progression of glioma, with a 5-year overall survival rate of 28.0% and a recurrence-free survival rate (RFS) of 20.0%. Based on median miR-873-5p expression, the patients were divided into the high and low expression groups. The 5-year overall survival rate and the RFS in the low expression group were remarkably lower than those in the high expression group (*p* < 0.05). According to the multivariate Cox regression analysis, tumor diameter, lymph node metastasis, TNM staging, and miR-873-5p were independent prognostic factors for the disease. See Figure 2 and Table 4.

### 3.3. miR-873-5p Could Inhibit the Proliferation and Invasion and Promote the Apoptosis of Glioma Cells

**Figure 3:**
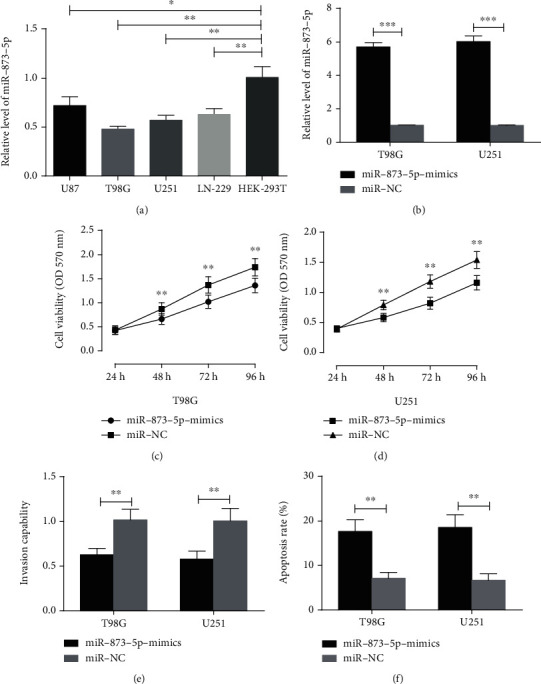
miR-873-5p expression in cells and its biological function to cells (a) miR-873-5p expression in HEK-293T cells is remarkably higher than that in U87, T98G, U251, and LN-229 cells. (b) The expression of miR-873-5p in the miR-873-5p-mimic group was remarkably higher than that in the miR-NC group in T98G and U251 cells transfected with miR-873-5p-mimics. (c, d) The proliferation ability of the miR-873-5p-mimic group of T98G and U251 cells was remarkably lower than that of the miR-NC group. (e) The cell invasion rate of the miR-873-5p-mimic group of T98G and U251 cells was remarkably lower than that of the miR-NC group. (f) The apoptosis rate of the miR-873-5p-mimic group of T98G and U251 cells was remarkably higher than that of the miR-NC group.∗ indicates *p* < 0.05, ∗∗ indicates *p* < 0.01, and ∗∗∗ indicates *p* < 0.001.

According to the detection, miR-873-5p expression in U87, T98G, U251, and LN-229 cells was remarkably lower than that in HEK-293T cells (*p* < 0.05). T98G and U251 cells were selected for the transfection. The expression was remarkably higher in the miR-873-5p-mimic group (*p* < 0.05). MTT assay showed that the proliferation was remarkably lower in the miR-873-5p-mimic group (*p* < 0.05). Transwell showed that the cell invasion rate was remarkably lower in the miR-873-5p-mimic group (*p* < 0.05). Flow cytometry showed that the apoptotic rate was remarkably higher in the miR-873-5p-mimic group (*p* < 0.05). See Figure 3.

## 4. Discussion

Many studies have found that the pathogenesis and progression of glioma are usually regulated by some genes, so understanding their regulatory mechanisms on the disease can help transform some of the mechanisms into corresponding therapeutic targets or markers for predicting the development and progression of glioma [17–19]. One of such regulatory genes is miRs. Some miRs cause tumor progression and invasion by regulating angiogenesis-related mechanisms, so changing tumor invasion by changing angiogenesis has become a possible therapeutic method [20]. According to Zhen et al., miR-449b-5p specifically binds to NEAT1 and then promotes the onset of glioma, so it is expected to become a potential target for its prognosis and treatment [21].

miR-873-5p, which was chosen for research in this study, is related to many cancers and plays an inhibitory role in some tumors. For instance, it inhibits colon cancer by inhibiting TUSC3/AKT signal transduction and inhibits rectal cancer progression via targeting ZEB1 to regulate epithelial-mesenchymal transition [22, 23]. In this study, miR-873-5p expression in OG was remarkably lower than that in CG, which suggests that this miR may be a potential indicator for diagnosing glioma. According to the ROC curves, the AUC of miR-873-5p for the diagnosis was 0.842, which indicates that miR-873-5p has a good diagnostic value for glioma. According to the analysis of the relationship between miR-873-5p and clinicopathological features, it was closely correlated with the tumor diameter, lymph node metastasis, and TNM staging of the patients. This demonstrates that miR-873-5p is possibly used to diagnose these clinical features. According to the ROC curves, the AUCs of miR-873-5p for diagnosing the features were 0.706, 0.865, and 0.793, respectively. Therefore, we may judge these clinical features by observing the expression of miR-873-5p. We collected the 5-year survival of the patients and found that the overall survival rate and the RFS in the low expression group were remarkably lower than those in the high expression group. The multivariate Cox analysis was conducted due to the poor prognosis of the patients. The results showed that tumor diameter, lymph node metastasis, TNM staging, and miR-873-5p were independent prognostic factors for the disease. Therefore, to observe these factors is beneficial to the management of high-risk patients.

Next, the effects of miR-873-5p on the cell biological functions were detected. According to Chen et al., the overexpression of miR-873 increases the apoptosis of cisplatin-resistant glioma cells and makes them sensitive to cisplatin-induced cell growth arrest and apoptosis [24], but effects of this miR on the biological functions are still unclear. In this study, miR-873-5p expression in four glioma cells was remarkably lower than that in normal cells, similar to the expression in the patients' serum. Additionally, compared with the miR-NC group, the cells in the miR-873-5p-mimic group had lower proliferation and invasion rates but a remarkably higher apoptotic rate. These findings reveal that miR-873-5p can inhibit glioma cells to proliferate and invade, and promote their apoptosis, so it is expected to be a therapeutic target for glioma.

There are still deficiencies in this study. First of all, the impact of miR-873-5p on the biological behavior of glioma cells was preliminarily explored, so the specific mechanism, the affected target genes, and the regulatory signaling pathways remain unclear. Secondly, tumor formation in nude mice was not carried out, so relevant research is needed to determine whether this miR can become a therapeutic target for glioma. Finally, the target gene or protein of this miR was not predicted. For example, Wnt/*β*-catenin and c-Met are target proteins and regulatory pathways in other cancers [25, 26]. Therefore, we hope to make up for these deficiencies in subsequent studies.

## 5. Conclusion

In summary, miR-873-5p can inhibit glioma cells to proliferate and invade, and promote their apoptosis, so it is expected to become a potential diagnostic index and therapeutic target for glioma.

## Figures and Tables

**Table 1:** Primer sequences.

Gene	Forward primers	Reverse primers
miR-873-5p	5′-ACACTCCAGCTGGGGCAGGAACTTGTGAG-3′	5′-TGGTGTCGTGGAGTCG-3′
U6	5′-CTCGCTTCGGCAGCACA-3′	5′-AACGCTTCACGAATTTGCGT-3′

**Table 2:** Relationship between miR-873-5p and pathological data.

Factors		Relative expression of miR-873-5p	*t* value	*p* value
Gender	Male (*n* = 26)	0.632 ± 0.058	0.147	1.473
	Female (*n* = 24)	0.607 ± 0.062		
Age	<55 years old (*n* = 14)	0.604 ± 0.047	1.684	0.099
	≥55 years old (*n* = 36)	0.637 ± 0.067		
Tumor diameter	<5 cm (*n* = 34)	0.670 ± 0.071	8.108	<0.001
	≥5 cm (*n* = 16)	0.508 ± 0.053		
Lymph node metastasis	Yes (*n* = 18)	0.524 ± 0.051	7.320	<0.001
	No (*n* = 32)	0.668 ± 0.074		
TNM staging	I+II (*n* = 32)	0.653 ± 0.065	6.638	<0.001
	III+IV (*n* = 18)	0.536 ± 0.049		

**Table 3:** ROC parameters.

Parameters	Disease diagnosis	Tumor diameter	Lymph node metastasis	TNM staging
AUC	0.842	0.706	0.865	0.793
Std. error	0.039	0.077	0.053	0.065
95% CI	0.766~0.917	0.556~0.856	0.762~0.968	0.667~0.920
Specificity	74.00%	58.82%	78.13%	71.88%
Sensitivity	80.00%	87.50%	83.33%	83.33%
Youden index	54.00%	46.32%	61.46%	55.21%
Cut-off	>0.744	<0.723	<0.657	<0.670

**Table 4:** Cox analysis.

Factors	Univariate Cox			Multivariate Cox		
	HR	*p* value	HR (95% CI)	HR	*p* value	HR (95% CI)
Gender (male vs. female)	2.758	0.458	1.337~3.522			
Age (<55 years old vs. ≥55 years old)	0.509	0.071	0.245~1.058			
Tumor diameter (<5 cm vs. ≥5 cm)	5.174	0.002	1.798~14.891	5.539	0.003	1.796~17.082
Lymph node metastasis (yes vs. no)	3.510	0.000	1.731~7.115	5.649	<0.001	2.405~13.268
TNM staging (I+II vs. III+IV)	0.382	0.008	0.188~0.777	0.573	0.166	0.260~1.260
Cystic degeneration of tumor (yes vs. no)	3.684	0.296	1.504~8.426			
Complicated with epilepsy (yes vs. no)	2.071	0.656	1.725~5.242			
miR-873-5p (high vs. low)	0.758	0.000	0.365~1.575	9.315	<0.001	3.108~27.914

## Data Availability

GSE103228 chip was selected.
